# Magnetic Cationic Liposomes-Based Delivery System Reduces Drug-Induced Cytotoxicity in an In Vitro Model of Hearing Loss

**DOI:** 10.3390/nano15191529

**Published:** 2025-10-07

**Authors:** Loredana Iftode, Camelia Mihaela Zara Danceanu, Anca Niculina Cadinoiu, Delia Mihaela Raţă, Marcel Popa, Luminița Labusca, Luminita Radulescu

**Affiliations:** 1 Faculty of Medicine, “Grigore T. Popa” University of Medicine and Pharmacy, 700115 Iasi, Romania; iftodeloredana@yahoo.com (L.I.); lmradulescu@yahoo.com (L.R.); 2 “Cristofor Simionescu” Faculty of Chemical Engineering and Environmental Protection, “Gheorghe Asachi” Technical University, 700050 Iasi, Romania; marcel.popa@guest.tuiasi.ro; 3 National Institute of Research and Development in Technical Physics, 700050 Iasi, Romania; cdanceanu@phys-iasi.ro; 4 “Ioan Haulica” Institute, Faculty of Medicine, “Apollonia” University of Iasi, 700511 Iasi, Romania; 5 Academy of Romanian Scientists, 050044 Bucharest, Romania; 6 Department of Orthopedics, “Sf. Spiridon” Emergency Clinical Hospital, 700111 Iasi, Romania

**Keywords:** magnetic liposomes, dexamethasone, drug-associated ototoxicity

## Abstract

Hearing loss is a major health burden, often caused by ototoxic drugs such as cisplatin and gentamicin. Effective therapy is limited by the poor penetrability of drugs into inner ear compartments. This study aimed to develop and test magnetic cationic liposomes as nanocarriers for targeted corticosteroid delivery to auditory hair cells. Carboxymethyl chitosan–coated liposomes were prepared by the lipid film hydration method, incorporating magnetic nanoparticles and dexamethasone phosphate in their aqueous core. The optimal liposomal formulation, in terms of size, zeta potential, and drug leakage over time, was selected and tested in an in vitro model of drug-induced ototoxicity. HEI-OC1 cells exposed to cisplatin or gentamicin were co-treated with the liposomal formulations, and viability, mitochondrial membrane potential, and β-galactosidase activity were assessed. The results demonstrated that magnetic, polymer-coated liposomes protected against cytotoxicity by preserving mitochondrial function and significantly reducing senescence. These findings provide a proof of concept for magnetically responsive liposomal systems as potential therapeutic platforms for preventing or treating drug-associated hearing loss.

## 1. Introduction

Hearing loss is a widespread condition affecting both pediatric and adult populations, with etiologies ranging from genetic mutations and environmental factors to iatrogenic causes such as ototoxic medications. According to the World Health Organization (WHO), more than 430 million people globally—more than 5% of the world’s population—suffer from disabling hearing loss, including approximately 34 million children. The prevalence of hearing impairment increases with age, impacting more than 25% of individuals older than 60 years [1,2].

Among the causes of acquired hearing loss, certain pharmacological agents are well-known for their ototoxic potential. Aminoglycoside antibiotics such as gentamicin, streptomycin, and tobramycin, as well as platinum-based chemotherapeutics like cisplatin and carboplatin, have been implicated in both temporary and permanent sensorineural hearing loss. Cisplatin, widely used in protocols for treating solid tumors, induces dose-dependent ototoxicity, with hearing loss reported in 23–50% of adults and up to 50–60% of children receiving the drug [3].

The pediatric population is particularly susceptible to drug-induced ototoxicity due to the ongoing development of their auditory systems, high relative drug dosages, and frequent exposure to ototoxic drugs during the treatment of cancer and severe infections. Hearing loss during early developmental stages can have profound consequences for language acquisition, academic performance, and psychosocial well-being. Therefore, effective preventive and therapeutic interventions are critical in pediatric care [4].

By employing cells, biomolecules, and biomaterials, regenerative strategies show promise for sensory restoration, including hearing recovery [5,6]. Efforts to differentiate stem cells into hair cell-like structures have yielded encouraging results [7], though these protocols are technically demanding, costly, and difficult to scale for broad clinical applications [8]. Consequently, maintaining and protecting native cochlear hair cells is a preferred strategy for mitigating the impact of ototoxic drugs [9].

However, therapeutic efficiency relies heavily on precise delivery to the inner ear. This is hindered by the cochlea’s anatomical and physiological barriers, including the blood–labyrinth barrier, the low permeability of drugs, and the potential for additional toxicity associated with invasive methods [10,11,12].

Nanotechnology-based delivery systems, particularly liposomes, have been explored to overcome these limitations. Liposomes are spherical vesicles composed of phospholipid bilayers, capable of encapsulating both hydrophilic and hydrophobic drugs. They are widely used in biomedical applications because of their biocompatibility and low toxicity [13,14,15]. Despite their advantages, conventional liposomes have limitations, such as moderate physical stability, complex production, and poor target specificity [16]. Furthermore, systemic administration often leads to the opsonization and protein corona formation, altering their pharmacokinetics and potentially reducing therapeutic efficacy [17].

Magnetic nanoparticles (MNPs), especially iron oxide-based, have gained attention for their excellent biocompatibility, biodegradability via endogenous iron metabolism, and imaging potential via magnetic resonance imaging (MRI). MNPs provide remote maneuverability under external magnetic fields, enabling controlled drug delivery and real-time tracking [18]. Mesenchymal stem cells (MSCs) incorporating MNPs have been shown to retain their biological phenotype and responsiveness to magnetic cues, allowing for targeted cell therapy and diagnostic applications such as magnetic particle imaging (MPI) [19]. Additionally, MNP-labeled adipose-derived stem cells demonstrated immunomodulatory effects, which could be leveraged to mitigate local inflammation [20].

Magnetic liposome hybrid systems that incorporate MNPs into liposomal structures have been obtained, aiming at targeted and traceable delivery. These platforms enhance drug release at specific sites when exposed to low-frequency alternating magnetic fields [21]. Such systems have been applied to otoprotection, as shown in studies where prednisolone-loaded magnetic liposomes were directed to the inner ear, significantly reducing cisplatin-induced hearing loss in murine models, outperforming intra-tympanic methylprednisolone [22]. Other corticosteroids, such as dexamethasone, have also demonstrated efficacy in protecting against cisplatin ototoxicity [23,24]. The present study focused on in vitro testing of a drug-induced ototoxicity model using a novel drug delivery carrier. These carriers consist of a magnetic liposomal system composed of: (i) an aqueous core encapsulating a hydrophilic corticosteroid (dexamethasone phosphate, Dex-P) and MNPs; (ii) a lipid bilayer formed by egg phosphatidylcholine (EPC), cholesterol (Chol), and the cationic lipid DOTAP; and (iii) an outer coating of carboxymethyl chitosan (CMCS), a biodegradable, biocompatible, and non-toxic polysaccharide. It is well known that liposomes are very advantageous platforms for the administration of active principles [25], but cationic liposomes containing DOTAP in their composition have demonstrated cytotoxic effects [26,27]. For these reasons, it was decided to mask the cationic liposomes with carboxymethyl chitosan. Also, the coating of liposomes with polymers was adopted to improve their colloidal stability both during storage and in vivo, as it is highlighted in different studies in the specialized literature. Previous studies have demonstrated that polymer coating increases the size of the vesicles and modifies the zeta potential values [28,29,30]. Polymer coating of liposomes has been adopted in various studies, where it has been shown to increase vesicle size and modify zeta potential values, thus having a significant impact on their stability. Importantly, such surface modifications improved pharmacokinetics, biodistribution, and reduced toxicity profiles [31,32]. The novelty of this study mainly consists in the use of a complex drug-loaded formulation based on lipids, a derivative of a natural polymer, and magnetic nanoparticles for the treatment of specific inner ear diseases. To the best of our knowledge, this type of system has not been tested before for such applications. In this context, we aimed at testing magnetic liposomal formulation as delivery methods for auditory hair cell rescue due to their ability to safely reach inner ear compartments. Combined with magnetic responsiveness, the use of magnetic liposomal carriers could translate into a safe, easy-to-use method for preventing or treating ototoxic drug-induced hearing loss. To evaluate the biological efficacy of the CMCS-coated magnetic liposomes, House Ear Institute-Organ of Corti 1 (HEI-OC1) cells were exposed to ototoxic drugs—cisplatin or gentamicin—and simultaneously treated with the Dex-P–encapsulating formulations. The study assessed cellular viability, mitochondrial membrane potential, and senescence-associated beta-galactosidase activity to determine the protective effects of the nanocarriers against drug-induced cytotoxicity.

## 2. Materials and Methods

### 2.1. Materials

1,2-Dioleoyloxy-3-trimethylammonium-propane chloride (Lipoid DOTAP) (DOTAP) and egg yolk phospholipids (Lipoid E PC S) (phosphatidylcholine content: ≥96%) (EPC) were purchased from Phospholipid GmbH, Köln, Germany. Cholesterol (CHOL), fluorescein disodium salt, and dexamethasone phosphate disodium salt (Dex-P) were purchased from Alfa Aesar. Carboxymethyl chitosan (CMCS) (carboxymethylation ≥ 80%), phosphate-buffered saline powder, and fetal bovine serum (FBS) were purchased from Sigma-Aldrich, St. Louis, MI, USA. Saline (NaCl, 9 mg/mL) was procured from B. Braun, Melsungen, Germany. Triton X and chloroform were obtained from VWR International LLC, Radnor, PA, USA. Iron (II) chloride tetrahydrate (FeCl_2_·4H_2_O) was purchased from Fluka. Pluronic F127, sodium hydroxide (NaOH), and iron (III) chloride anhydrous originated from Lachner.

### 2.2. Preparation Method of Magnetic Cationic Liposomes Coated with Carboxymethyl Chitosan

Liposomes were obtained from egg yolk phospholipids (EPC), 1,2-dioleoyloxy-3-trimethylammonium-propane chloride (DOTAP), and cholesterol (CHOL) in different molar ratios using the thin lipid film hydration method, as detailed in a previously published study [32]. The first step was to obtain a lipid film. Briefly, the lipids were dissolved in an organic solvent (chloroform) in a 250 mL round-bottom flask. To obtain the lipid film, chloroform was removed under reduced pressure using a Heidolph rotary evaporator (Hei-VAP ML Heidolph Instruments GmbH & Co.KG, Schwabach, Germany) at a rotation speed of 160 rpm and a temperature of 40 °C. Finally, the lipid membrane was kept under constant argon flow for 20 min to dry completely. The second step was the hydration of the lipid film and the obtaining of lipid vesicles. For this, 12.5 mg magnetic nanoparticles (MNPs) were dispersed by ultrasonication in the hydration solution (5 mL phosphate buffer solution with pH 7.4, at 40 °C) to be added over the obtained lipid film. Magnetic nanoparticles were prepared by the co-precipitation method, as described comprehensively in our previous studies [33,34,35]. Multilamellar vesicles were obtained after vigorous shaking using a DLab MX-S Variable Speed Vortex. The resulting vesicle suspension was then maintained at 40 °C in a bath sonicator for 30 min to reduce the size and obtain a homogeneous population of liposomes. Magnetic liposomes were separated from non-encapsulated MNPs by centrifugation (10,000 rpm) for 15 min, using a Hettich UNIVERSAL 320 centrifuge with the 12-place Clip Rotor. The retention degree of MNPs in the aqueous core of the liposomes was then quantified. Thus, to 0.5 mL of magnetic liposome suspension, 0.5 mL of Triton X-100 (2% aqueous solution) was added to lyse the lipid membrane and release the MNPs. After vigorous vortexing, the MNPs were separated by centrifugation. To remove the lipids and then the detergent, one additional wash with a detergent solution was followed by four more distilled water washes. Each wash was followed by centrifugation and separation of the supernatant. After the final centrifugation, the MNPs were separated and placed in a vacuum oven at 50 °C until a constant mass was achieved. The quantification of the retention degree of MNPs was conducted in triplicate.

The corticosteroid drug (Dex-P) was encapsulated in liposomes during the lipid film hydration process. Dex-P was dissolved in a phosphate buffer solution before adding the MNPs. The unencapsulated drug was removed by dialysis using a tubular cellulose membrane (12,000–14,000 Da) in ultrapure water until the drug was no longer detected spectrophotometrically in the washing water.

The third step in preparing complex systems involves coating magnetic liposomes with CMCS. First, 2 mL of a 1% *w*/*v* carboxylate chitosan solution in ultrapure water was added dropwise to 2 mL of liposome suspension under magnetic stirring at room temperature. Then, for 2 h, the suspension was kept under stirring to achieve the electrostatic attraction of CMCS to the positive surface of the liposomes [32,33]. Table 1 presents the experimental program for the preparation of the liposomes.

### 2.3. Characterization of CMCS-Coated Magnetic Liposomes

#### 2.3.1. Morphology of Liposomes

The average diameter of the obtained liposomes was determined by dynamic light scattering (DLS), at a concentration of 1% (*w*/*v*) in ultrapure water, at 25 °C. The zeta potential was measured at the same temperature by Doppler laser micro-electrophoresis after dilution in a saline solution at an appropriate counting rate. For these two analyses, Zetasizer Nano ZS equipment from Malvern Panalytical Limited, Worcestershire, UK, was used.

TEM analysis was used to determine the shape and size of the obtained liposomal drug carriers. A drop of diluted liposome dispersion was placed on a TEM grid and allowed air dry before analysis. The grid was observed with a transmission electron microscope from PHILIPS CM100, Amsterdam, Netherlands, operating at an accelerated voltage of 100 kV, with a magnification of 19,000. The image analysis was performed using a software Visilog 5.0 from Noesis, France.

#### 2.3.2. FT-IR Spectroscopy

Fourier-transform infrared (FT-IR) spectroscopy was used to analyze the structure of the CMCS-coated magnetic liposomes. The liposome samples, dried by lyophilization, were analyzed using a Shimadzu IRSpirit spectrometer equipped with a QATR-S single reflection ATR accessory. FT-IR spectra were recorded at room temperature in the range 400–4000 cm^−1^ with a resolution of 16 cm^−1^, in absorbance mode. LabSolutionsIR software Version 2.30 was used for data analysis.

#### 2.3.3. Stability Tests

The stability of magnetic liposomes was monitored for one month after storage in a phosphate buffered saline (PBS) solution with a pH of 7.4 at 4 °C (refrigerator) and room temperature (25 ± 2 °C), in the dark. Then, 20 μL of liposome suspension from tightly closed containers was periodically withdrawn and diluted with 4 mL of PBS to determine spectrophotometrically the drug leakage from CMCS-coated and uncoated magnetic liposomes. Experiments were performed in triplicate.

To better simulate in vitro conditions, the stability of CLDM-3 and CLDMC-3 formulations over time was monitored in 10% FBS (Sigma-Aldrich) medium at 37 °C, focusing on parameters such as particle size, PDI value, and zeta potential. To evaluate these parameters, Zetasizer Nano ZS equipment from Malvern Panalytical Limited, Worcestershire, UK, was used. For comparison, these parameters were also monitored at 4 °C as well as in two other media, ultrapure water and weak ionic medium (1 mM NaCl solution): 250 µL of liposomes suspension was added to 4 mL of test solution (ultrapure water, weak ionic solution or 10% FBS solution) and maintained at 4 °C and 37 °C, respectively. At the predetermined times, the parameters mentioned above were analyzed, taking into account the concentrations required for each type of analysis. Experiments were carried out in triplicate.

#### 2.3.4. Drug Encapsulation and Release Efficiency

The encapsulation efficiency of Dex-P was assessed after prior disruption of the liposomes in the presence of a surfactant (Triton X-100) by a spectrophotometric method using a NanoDrop™ One Microvolume UV–vis Spectrophotometer, ThermoFisher Scientific, Madison, WI, USA. Prior to spectrophotometric analysis, the solution was centrifuged at 10,000 rpm for 15 min using a Hettich UNIVERSAL 320 centrifuge to mitigate lipid absorption. The calibration curve of Dex-P in PBS solution at a wavelength of 242 nm was used to calculate the amount of drug remaining in the aqueous core of the liposomes.

The release degree of Dex-P from liposomes was monitored at 37 °C in a PBS solution with a pH of 7.4 to simulate the physiological environment. An Agilent 708-DS dissolution system (Agilent Technologies LDA, Bayan Penang, Malaysia) equipped with an 850-DS sampling station was used for this experiment. Briefly, a suspension of drug-loaded liposomes (2 mL) was introduced into sealed cellulose dialysis bags (12 to 14 kDa), and immersed in 200 mL of PBS buffer at 37 °C in a water bath, under gentle agitation (50 rpm). The concentrations of Dex-P released at predetermined times in the receiving medium were evaluated by UV–vis spectroscopy.

#### 2.3.5. Magnetic Properties

The magnetic properties of the lyophilized CMCS-coated magnetic liposomes (coercivity—Hc; rest magnetization—Mr; saturation magnetization—Ms) were evaluated by vibrating sample magnetometry (VSM) (MicroMag 3900 Series VSM, Princeton Measurements Corporation, Princeton, NJ, USA) at room temperature. A Labconco™ FreeZone™ Benchtop Freeze Dryer (Kansas City, MO, USA) (2.5 L, −50 °C) was used to lyophilize the CMCS-coated magnetic liposomes.

### 2.4. In Vitro Studies of HEI-OC1 Cell Cultures

For in vitro tests, formulations based on MNPs and cationic liposomes without or with MNPs, uncoated or coated with CMCS, unloaded or loaded with drug were tested, as follows: (i) magnetic nanoparticles (MNPs), (ii) cationic liposomes (CL), (iii) magnetic cationic liposomes (CLM), (iv) cationic CMCS-coated liposomes (CLC), (v) CMCS-coated magnetic cationic liposomes (CLMC), (vi) cationic liposomes loaded with Dex-P (CLD), (vii) magnetic cationic liposomes loaded with Dex-P (CLDM), (viii) CMCS-coated cationic liposomes loaded with Dex-P (CLDC), (ix) CMCS-coated magnetic cationic liposomes loaded with Dex-P (CLDMC). Biological characterization was conducted on liposomal formulations obtained with the mass ratio of 19/4/2 between EPC/Chol/DOTAP lipids, considering that samples with this composition were evaluated as optimal in terms of size, zeta potential, and drug leakage over time. Liposomes without MNPs in their core have been previously characterized, and the results are presented in detail in the manuscript published by Iftode L. et al. [32].

#### 2.4.1. Cells

The House Ear Institute-Organ of Corti 1 (HEI-OC1) cell line was obtained as a kind gift from Professor Kalineck’s laboratory [36]. Cells were initially cultured under proliferative (permissive) conditions in a humidified incubator at 33 °C with 10% CO_2_, using T25 adherent culture flasks. The culture medium consisted of Dulbecco’s Modified Eagle Medium (DMEM) supplemented with 10% fetal calf serum (FCS), and no antibiotics were added to ensure optimal preservation of cellular physiology and function.

#### 2.4.2. Liposomes’ Biological Characterization

##### Cell Viability Tests

For the cytotoxicity, HEI-OC1 from passages 4–5 was plated in 96-well plates at 1 × 10^4^ cells/well and incubated for 48 h. Liposomes of each type were first added to the cell culture medium at two concentrations of 12.5 µg/mL and 25 µg/mL and incubated for another 48 h. A second test was performed at two other concentrations of 6.25 µg/mL and 12 µg/mL to find the optimal concentration at which cells show good viability in the presence of liposomal formulations. The reported concentrations (6.25 µg/mL, 12 µg/mL, 12.5 µg/mL, and 25 µg/mL) refer to total lipid content. All tests were carried out in triplicate. The colorimetric assay with 5-dimethylthiazol-2-yl-2, 5-diphenyltetrazolium bromide (MTT) (the Vybrant MTT cell proliferation assay from ThermoFisher Scientific) was conducted according to the supplier’s instructions, using dimethylsulphoxide (DMSO) as the dilution agent. Absorbance was read at 570 nm (Synergy HTX Multi-Mode Reader-Biotech). Cell viability (CV) was obtained using the formula CV = 100 × (ODs − ODb)/(ODc − ODb), where ODs = liposome-treated cell OD; ODb = blank (media only) OD; and ODc = untreated cell optical density (OD). Further rescue experiments were performed at the lower concentrations 6.25 µg/mL and 12 µg/mL, which were determined to be non-toxic. Absorbance was read at 570 nm.

##### Gentamicin and Cisplatin Toxicity

An MTT assay with increasing concentrations of gentamicin (genta) and cisplatin (cis) was performed after HEI-OC1 cells from passages 4–5 were plated in 96-well plates, at 1 × 10^4^ cells/well, and incubated for 48 h. This was followed by treatment at increasing concentrations of genta (2, 8, 14, and 20 μg/mL) and cis (1, 4, 7, and 10 μg/mL). Cell viability was evaluated after 48 h of cell-driven interaction.

##### HEI-OC1 Recovery After Gentamicin/Cisplatin Exposure and Treatment with Liposomal Formulations

As in the other two assays presented above, HEI-OC1 cells from passages 4–5 were plated in 96-well plates, at 1 × 10^4^ cells/well, and incubated for 48 h. In this case, genta (20 µg/mL) and cis (4 µg/mL), respectively, together with liposomal formulations (6.25 and 12 µg/mL), were added to the cell culture medium and incubated for an additional 24 h. The same colorimetric MTT method was used to assess cell viability. Absorbance was read at 570 nm.

##### Mitochondrial Membrane Potential—JC-1

For the detection of mitochondrial membrane potential (MMP) in HEI-OC1 cells the 5,5,6,6’-tetrachloro-1,1’,3,3’tetraethylbenzimi-dazoylcarbocyanine iodide (JC-1) assay was used as follows. The HEI-OC1 cells cultured in a 24-well plate at a density of 1 × 10^5^ cells/mL for 24 h were treated with 12 µg/mL liposomal formulation and co-treated with 10 or 20 µg/mL cisplatin/gentamicin for another 24 h. In the following steps, the cells were stained with 10 µg/mL of JC-1 and incubated at 37 °C for 20 min. MMP was qualitatively assessed using an inverted fluorescent microscope (EVOS Life Science, Waltham, MA, USA). Quantitative fluorescence was assessed with ImageJ software in at least five images/samples. Distinct quantification of green and red fluorescence was performed. The results are presented as red/green fluorescence intensity. Images were opened in ImageJ Version 13.0.6 as a stack and converted to 8-bit grayscale. Background noise was removed using the “Subtract the Background” tool. Fluorescence intensity was quantified by setting the measurement to “integrated density” and using the “measure stack” function. Data were exported in CSV format and analyzed in Excel for red/green fluorescence ratios and percentage calculations relative to controls. Absorbance was read at 570 nm.

##### Quantitative Assessment of Iron Nanoparticles’ Uptake Using Ferrozine Assay

For these assessments, HEI-OC1 cells were plated in 24-well plates at 1 × 10^5^ cells/well. After 24 h, MNPs (20 µg/mL) and liposomes in different formulations (CL, CLC, CLM, CLMC, CLDM, and CLDMC) (12 µg/mL) were added to the complete medium. After 48 h, cells were washed twice with PBS to remove the extracellular MNPs, then fixed in 70% ethanol for 15 min and again washed in PBS three times for 5 min. Then, 500 µL of NaOH (50 mM) was added to 3 wells per experiment and kept for 2 h on a shaking plate. Aliquots of cell lysates were then transferred to 1.5 mL Eppendorf tubes and mixed with 500 µL of 10 mM HCl, and 500 µL of iron-releasing reagents (a freshly mixed solution of equal volumes of 1.4 M HCl and 4.5% (*w*/*v*) KMnO_4_) (Merck, Germany) in distilled H_2_O. These mixtures were incubated for 2 h at 60 °C in a fume hood before 150 µL of iron-detection reagent (6.5 mM ferrozine, 6.5 mM neocuproine, 2.5 M ammonium acetate, and 1 M ascorbic acid dissolved in water) was added to each tube. The reagents used were purchased from Sigma-Aldrich. After 30 min, the absorbance was read at 570 nm. A calibration curve was set up using FeCl_3_ standards (0–300 µM) in 10 mM HCl. For counting, cells previously fixed with ethanol 80% for 10 min were treated with diamidino-2-phenylindole (DAPI, ThermoFisher Scientific) 0.1 µg/mL for 5 min and washed twice. Cell count/well (x) was performed using a fluorescent microscope and a grid using the following formula: x = 2 ∗ A/(L ∗ L) ∗ N where A is the surface of the well, L is the size of one square in the grid, N is the number of cells per region of interest (ROI), at least five ROI per sample.

##### Beta-Galactosidase Activity (B-Gal)

Cells were seeded in 96-well plates at a density of 1 × 10^4^ cells per well. After 24 h, liposomal formulations were added to obtain a concentration of 12 µg/mL. Beta-galactosidase enzyme activity was detected using the Abcam β-galactosidase (B-Gal) Detection Kit (Fluorometric), following the manufacturer’s instructions. Briefly, after 48 h of treatment, cells were lysed through four freeze–thaw cycles. Fluorescein di-β-D-galactopyranoside (FDG) was then added, and the cells were incubated for another 1 h at 37 °C. Subsequently, a stop buffer solution was added, and fluorescence was measured with a microplate reader (excitation/emission 490–550 nm). Cell counting was performed at each time point from separate wells after trypsinization using a cell counter (using the above protocol).

##### Statistical Analysis

The experiments were conducted in triplicate, and statistical analyses were carried out (using GraphPad 10.4.2). The statistical significance of the results obtained from the stability tests was analyzed using one-way ANOVA with the Tukey post hoc test. Experimental results with *p* values < 0.05 were considered statistically significant. Data are presented as mean ± SD (*n* = 3 independent experiments). Group differences were analyzed by one-way ANOVA followed by Bonferroni post hoc multiple comparisons for (i) β-gal activity vs. drug-only controls and (ii) JC-1 red/green ratios vs. drug-only controls within each drug condition (cisplatin, gentamicin). Significance was set at two-tailed α = 0.05 after adjustment. Exact adjusted *p*-values are reported in the figure/table annotations where significant; otherwise comparisons are denoted ns (*p* ≥ 0.05).

## 3. Results

### 3.1. Characterization of CMCS-Coated Magnetic Cationic Liposomes

#### 3.1.1. Morphology of Liposomes

The intensity-weighted hydrodynamic diameters, polydispersity index (PDI), zeta potential values, the loading efficiencies of MNPs and drug, as well as the final composition (mg/mL of each component) of uncoated and CMCS-coated magnetic cationic liposomes are presented in Table 2. The average diameter of uncoated magnetic liposomes was between 207 and 212 nm. Coating magnetic liposomes with CMCS led to an increase in their size, with values ranging between 268 and 306 nm.

**Figure 1:**
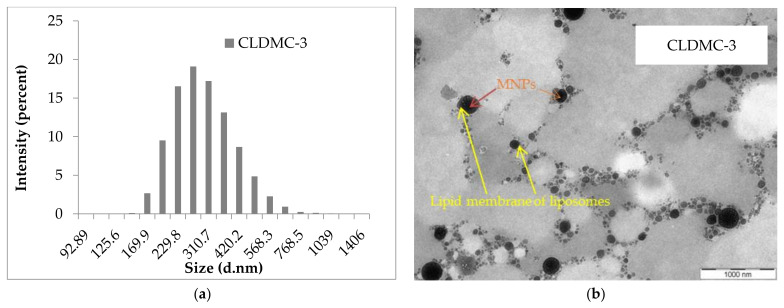
(**a**) The intensity-weighted size distribution and (**b**) TEM image of CLDMC-3 sample (CMCS-coated magnetic liposomes) obtained at an accelerating voltage of 100 kV with a magnification of 19,000.

The size distribution in ultrapure water and the TEM image of sample CLDMC-3 (CMCS-coated magnetic liposomes) are shown in Figure 1. For MNPs, these results are presented in Figure S1 (in Supporting Information). As can be seen, the size distribution curve of the CLDMC-3 sample is monomodal with particle populations ranging between 160 and 600 nm (Figure 1a), and their shape is spherical (Figure 1b).

Figure 1b shows a TEM image of CMCS-coated magnetic liposomes at a magnification of 19,000×. Spherical liposomes, with sizes ranging from 160 to 600 nm (as shown by the DLS analysis in Figure 1a), appear as light structures, while encapsulated magnetic nanoparticles, with dimensions of 10–18 nm, are identifiable as dark electron-dense spots. The polymer coating, evidenced by the increase in liposome size and the change in zeta potential, cannot be clearly distinguished in the TEM image at this resolution. Arrows are used to indicate representative magnetic nanoparticles and liposome outlines.

The result obtained from the DLS analysis for the CLDMC-3 sample can be correlated with the image obtained by TEM analysis.

The zeta potential for uncoated liposomes in saline solution (0.9%) has positive values ranging between 14 and 18 mV, as can be seen in Table 2. DOTAP confers positive zeta potential. Due to the carboxyl groups present in CMCS, coating the liposomes with this chitosan derivative led to a change in zeta potential values, from positive to negative values (Table 2).

CMCS-coated magnetic liposomes displayed spherical morphology and increased size compared to uncoated systems, confirming successful coating and encapsulation of magnetic nanoparticles.

#### 3.1.2. FT-IR Spectroscopy

**Figure 2:**
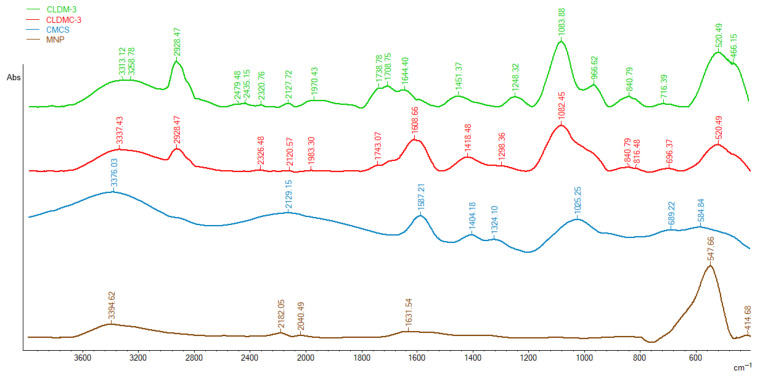
FT-IR spectra of the CMCS, MNPs, magnetic liposomes (CLDM-3), and coated magnetic liposomes (CLDMC-3).

Infrared spectroscopy (FT-IR) was used to confirm the coating of magnetic cationic liposomes with the CMCS and the presence of magnetic nanoparticles in the structure of liposomes. Figure 2 shows the FT-IR spectra for magnetic nanoparticles (MNPs), carboxylated chitosan (CMCS), liposomes containing magnetic nanoparticle (CLDM-3), and magnetic liposomes coated with CMCS (CLDMC-3).

In the FT-IR spectrum of liposomes (Figure 2), several characteristic peaks can be observed, namely: the peak at 1248 cm^−1^ in the CLDM-3 spectrum and 1298 cm^−1^ in the CLDMC-3, respectively, can be attributed to the antisymmetric PO^2−^ stretch and the one at approximately 1740 cm^−1^ to the [C=O] stretch of EPC; the broad peak at approximately 1450 cm^−1^ can correspond to hexagonal packing of acyl chains and the band at 966 cm^−1^ can be attributed to the stretch of the asymmetric terminal choline group. In the regions 716 cm^−1^ and 1083 cm^−1^, other characteristic peaks of egg phosphatidylcholine are located [37].

FT-IR spectra of CMCS (Figure 2) show a broad peak at approximately 1587 cm^−1^ characteristic of the asymmetric carboxylate group (COO^−^) and at 1404 cm^−1^, the symmetric carboxylate group (COO^−^) [38].

The successful coating of magnetic liposomes is revealed by the appearance or the shift of characteristic peaks in the FT-IR spectrum of the magnetic liposomes coated with CMCS (CLDMC-3) (Figure 2) sample as follows:

(i) Appearance in the spectrum of CLDMC-3 sample of peaks at 2928 cm^−1^, 1082 cm^−1^, and 840 cm^−1^ characteristic of the uncoated magnetic liposomes sample;
(ii) The shift of the peaks from 3376 cm^−1^ to 3337 cm^−1^, from 1587 cm^−1^ to 1608 cm^−1^, and from 1404 cm^−1^ to 1418 cm^−1^ in the spectrum of CMCS to spectrum of CLDMC-3;
(iii) The presence of the characteristic CMCS peak at 1025 cm^−1^ in the broad peak of the coated magnetic liposomes.

Finally, the FT-IR spectrum of magnetic nanoparticles shows an absorption peak at approximately 547 cm^−1^ that corresponds to the vibration of the Fe-O bonds in Fe_3_O_4_. Also, an absorption band was identified around 3394 cm^−1^, which can be attributed to the stretching vibration of the hydroxyl groups on the surface of the MNPs [35]. In Figure 2, we can observe in the FT-IR spectra of samples CLDM-3 and CLDMC-3 a shift of the peak characteristic for the Fe-O bonds from 547 cm^−1^ to around 520 cm^−1^, which reveals the presence of magnetic nanoparticles in the two analyzed samples.

#### 3.1.3. Stability Tests

**Figure 3:**
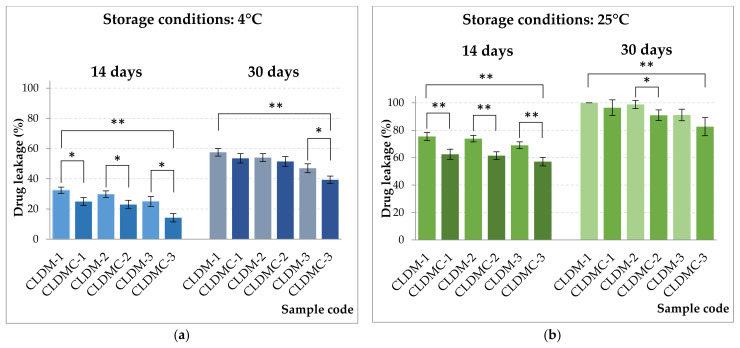
Drug leakage from magnetic liposomes uncovered and covered with CMCS stored at two different temperatures: (**a**) 4 °C and (**b**) 25 ± 2 °C. Data presented as mean ± SD, *n* = 3. Statistical significances are marked (* *p* < 0.05, ** *p* < 0.01).

The obtained magnetic cationic liposomes, uncoated and coated with CMCS, were stored at two different temperatures (4 °C and 25 ± 2 °C), in PBS medium with pH = 7.4 and in the dark. After 14 and 30 days, drug leakage was evaluated spectrophotometrically, and the results obtained are represented in Figure 3. Drug leakage after 14 days ranged between 14 and 32% for samples stored in the refrigerator and between 57 and 75% for samples stored at room temperature. Also, after 30 days, the drug leakage varied between 39 and 58% for the samples stored in the refrigerator and between 83 and 100% for the samples stored at room temperature, which indicates that their stability is higher at low temperatures. CMCS coating increased liposome stability at both storage temperatures. Polymer coating improved the stability of magnetic liposomes, reducing drug leakage, especially under refrigerated conditions.

**Figure 4:**
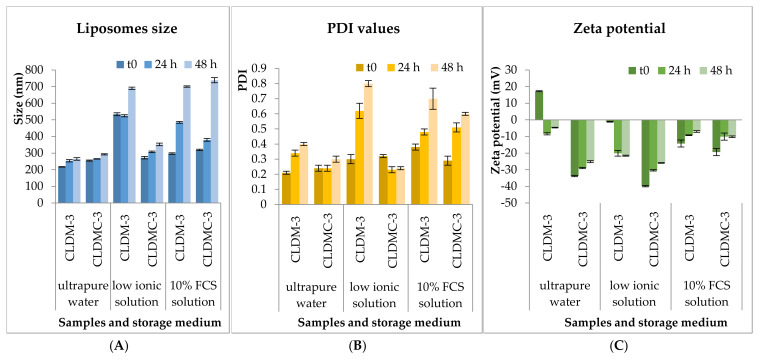
CLDM-3 and CLDMC-3 liposomes’ size (**A**), PDI value (**B**), and zeta potential (**C**) evaluated at 37 °C.

The stability of CLDM-3 and CLDMC-3 formulations was monitored for 48 h under simulated in vitro conditions, in 10% FCS medium (Sigma-Aldrich), as well as in the other two media for comparison, ultrapure water and weak ionic medium (1 mM NaCl solution), at 37 °C. Liposomes’ size, PDI value, and zeta potential were evaluated and the results obtained are presented in Figure 4.

**Figure 5:**
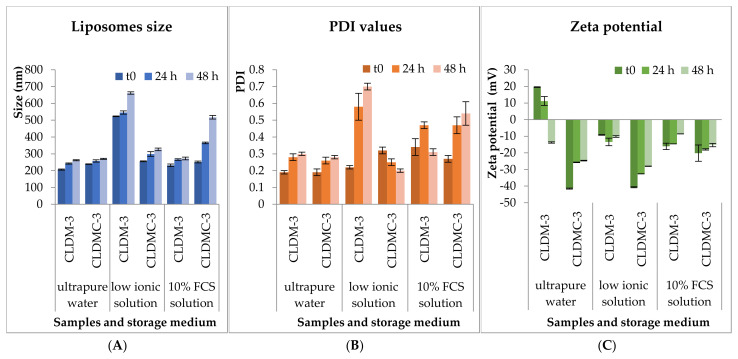
CLDM-3 and CLDMC-3 liposomes’ size (**A**), PDI value (**B**), and zeta potential (**C**) evaluated at 4 °C.

The results obtained at 4 °C are presented in Figure 5.

#### 3.1.4. Drug Encapsulation and Release Efficiency

The drug encapsulation efficiency for uncoated and CMCS-coated magnetic liposomes is presented in Table 2. It can be seen that the percentage of Dex-P found in uncoated liposomes after their destruction, compared to the amount of drug in the solution used to hydrate the lipid film, was between 8.4 and 9.2%, while the loading efficiency of Dex-P in CMCS-coated liposomes decreased after the coating step and was between 6.5 and 7.7%. This decrease in Dex-P loading efficiency was due to the release of the drug into the aqueous medium during the 2 h of CMCS coating of the magnetic liposomes, which occurred at room temperature and under stirring.

**Figure 6:**
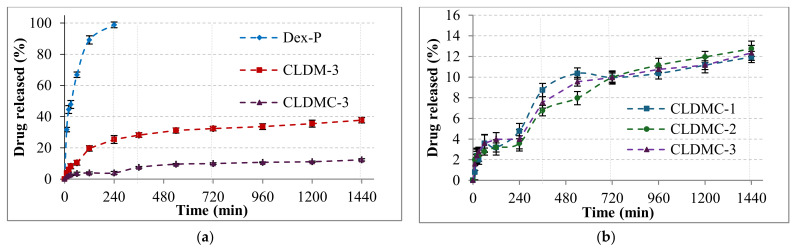
Drug release kinetic curves for: (**a**) free Dex-P and CLDM-3 (magnetic liposomes) and CLDMC-3 (magnetic liposomes coated with CMCS) samples, and (**b**) magnetic liposomes coated with CMCS (CLDMC-1, CLDMC-2, and CLDMC-3 samples) for 24 h. The data are presented as mean ± SD, *n* = 3.

In vitro drug release was evaluated at 37 °C in phosphate-buffered saline (PBS) at pH 7.4, assuming similar pH conditions in the human body [39]. The release curves for Dex-P from the tested liposomal formulations are shown in Figure 6. The liposome suspensions, having similar amounts of drug encapsulated, were first introduced into dialysis bags, and then the well-sealed bags were introduced into a 200 mL PBS solution in the recipient vessel. The same amount of drug as that found in the liposomes was used as a control to highlight the release curve of free drug through the dialysis membrane.

As can be seen in Figure 6a, the free Dex-P crossed the dialysis membrane in a proportion of approximately 100% after 4 h, while the drug encapsulated in magnetic liposomes (CLDM-3) was released in a proportion of 25%, and that from magnetic liposomes coated with CMCS (CLDMC-3) was only 4% in this time interval. There was no significant difference between the three tested samples after 24 h of monitoring the release of the corticosteroid drug. CMCS-coated formulations showed slightly reduced encapsulation efficiency but enabled a slower, more sustained release of dexamethasone compared to uncoated liposomes.

#### 3.1.5. Magnetic Properties

**Figure 7:**
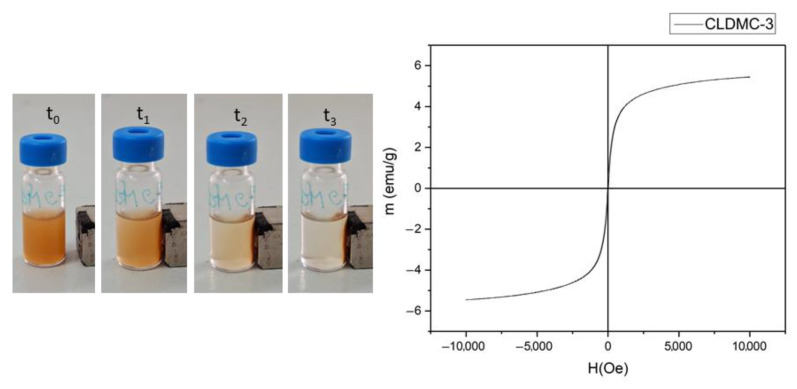
The CLDMC-3 sample suspension in PBS in presence of magnetic external field (t_0_ = 0; t_1_ = 10 s; t_2_ = 60 s; t_3_ = 120 s) (**left**) and the magnetization curve (**right**).

An external magnetic field was applied to the magnetic liposome suspension, and their behavior was monitored for 2 min. The images recorded at different times for the CLDMC-3 sample are shown in Figure 7, and the effect of the external magnetic field can be easily observed by the migration of the liposomes incorporating MNPs towards the magnet. The magnetic properties of the CLDMC-3 liposome sample were also determined by a vibrating sample magnetometer (VSM) at room temperature. From the magnetization curve of the analyzed sample, shown in Figure 7, it can be observed that the saturation magnetization value (Ms) was approximately 5.5 emu/g, and the coercivity (Hc) and remanence (Mr) were both zero, which proves the superparamagnetism of CMCS-coated magnetic liposomes [40]. CMCS-coated magnetic liposomes demonstrated superparamagnetic behavior, validating their potential for external magnetic guidance.

### 3.2. Liposomes’ Biological Characterization

#### 3.2.1. HEI-OC1 Viability

**Figure 8:**
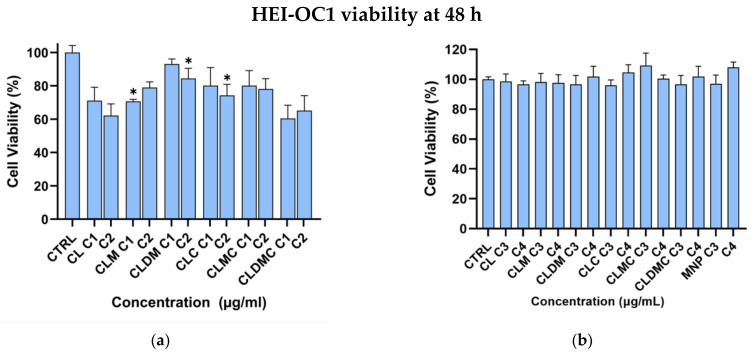
Cell viability of HEI-OC1 after 48 h in the presence of liposomal formulations: (**a**) C1 = 12.5 µg/mL and C2 = 25 µg/mL; (**b**) C3 = 6.25 µg/mL and C4 = 12 µg/mL. Data are represented as mean and SD, *n* = 3; * *p* < 0.05, compared with the control group.

The MTT assay is a colorimetric assay widely used to assess cell viability, proliferation, and drug-related cytotoxicity. Metabolically active cells reduce MTT into formazan crystals via mitochondrial NAD(P)H-dependent oxidoreductases while impaired mitochondrial activity fails to reduce MTT, which correlates with the number of metabolically active, viable cells. After 48 h of interaction between cells and the first two liposome concentrations, 12.5 and 25 μg/mL (Figure 8a), the highest cell viability was observed for CLDM at a tested concentration of 12.5 μg/mL (93.01% of untreated controls), while the lowest viability was recorded for CLDMC using 12.5 μg/mL.

Given this, a new test was performed, decreasing the liposome concentration, to detect a functional concentration of liposomes that could be added to the culture medium without causing cytotoxicity. Thus, at liposome concentrations of 6.25 μg/mL and 12 μg/mL (Figure 8b), cell viability was in the range 93–114%, motivating our choice to keep the latter as the working concentration for further rescue studies. The concentrations (6.25 μg/mL, 12 μg/mL, 12.5 μg/mL, and 25 μg/mL) represent the total lipid content. At lower concentrations, liposomal formulations preserved cell viability, with magnetic nanoparticle-containing carriers showing the best tolerance.

##### Cytotoxic Effect of Gentamicin and Cisplatin on HEI-OC1 Cells

**Figure 9:**
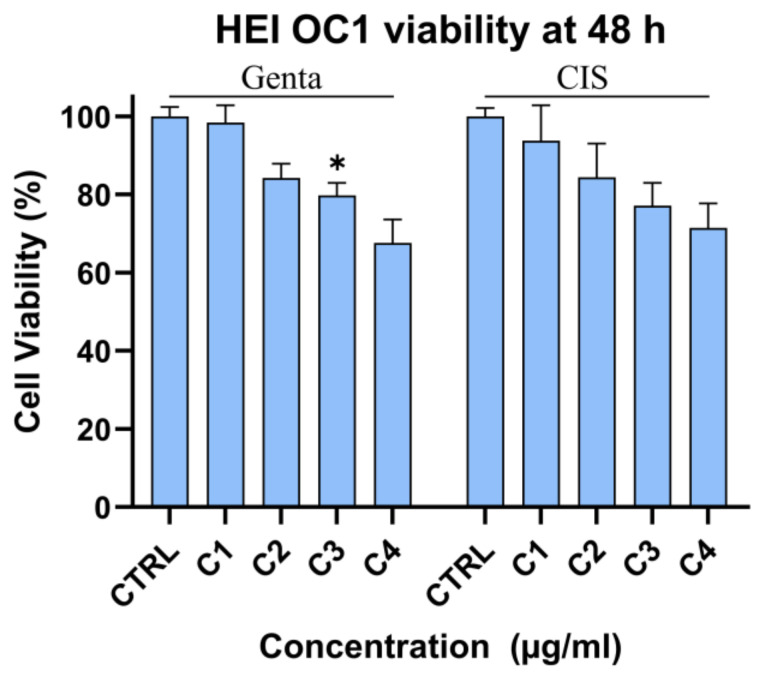
Cell viability assessment by an MTT assay. HEI-OC1 cells were treated with the designated concentrations of gentamicin (C1 = 2, C2 = 8, C3 = 14, C4 = 20 μg/mL; left) and cisplatin (C1 = 1, C2 = 4, C3 = 7, C4 = 10 μg/mL; right) for 48 h, *n* = 3, * = statistically significant; *p* ≤ 0.05.

To investigate the effect of gentamycin (genta) and cisplatin (cis) on HEI-OC1, cells were exposed to increasing concentrations of the respective drugs. Genta (2, 8, 14, and 20 μg/mL) and cis (1, 4, 7, and 10 μg/mL) were added to the culture media. After 48 h, the MTT assay revealed that drugs affected cell viability under these experimental conditions in a concentration-dependent manner. Significant decrease in HQI-OC1 viability was observed starting from 14 μg/mL for genta and from 7 µg/mL for cis (84.3% and 84.38% respectively). Cell viability decreased with increased concentration of drug to 67.5 and 71.43% of non-treated controls, respectively. Given the results, a concentration of 20 μg/mL for genta and 7 μg/mL for cis was used for further tests (Figure 9).

The MTT assay demonstrated that gentamicin and cisplatin treatment decreased cell viability in a concentration-dependent manner. Both drugs induced dose-dependent cytotoxicity in HEI-OC1 cells, with cisplatin showing stronger toxicity at lower concentrations.

##### Liposomal Formulation Uptake Based on Iron Content (Ferrozine Assay)

**Figure 10:**
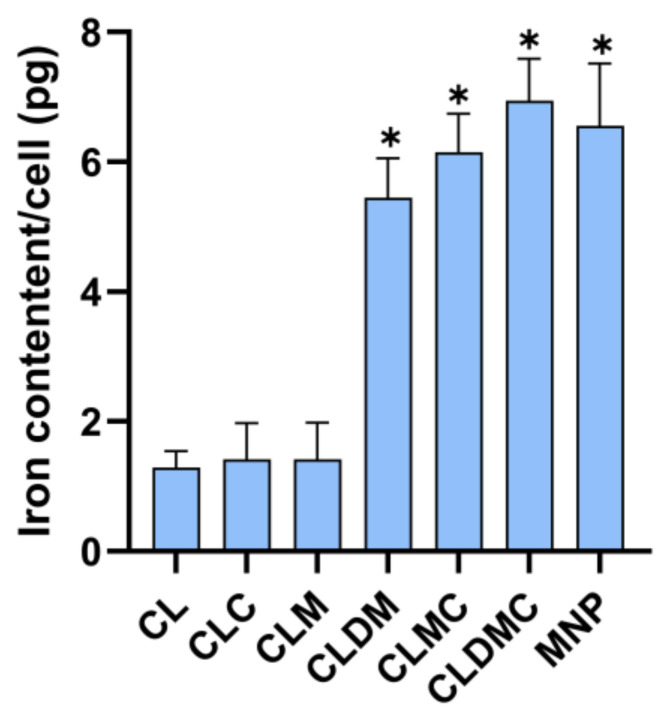
Iron taken up (in picograms) by HEI-OC1 cells after 48 h of liposome interaction (12 µg/mL) as determined by the ferrozine assay, * = statistically significant; *p* ≤ 0.05.

The test investigated the amount of iron-containing liposomes that could be taken up in single HEI-OC1 cells in 48 h of cell–liposome contact when a liposome concentration of [12 µg/mL] was added to the culture media without cell uptake enhancers. Specifically, the highest iron content per cell (6394 pg) was observed for CLDMC, with no statistically significant differences compared to CLM, CLDM, and free MNPs. As expected, all MNP-loaded liposomes showed significantly higher iron uptake than the MNP-free formulations (CL and CLD), which were included for reference as controls (Figure 10). HEI-OC1 cells internalized all MNP-containing formulations, with the highest uptake for CMCS-coated magnetic liposomes.

##### Effect of Liposomal Formulation Treatment on HEI-OC1 Cells Exposed to Drug Toxicity—Cell Viability Using MTT

In the next step, cells exposed to ototoxic drugs (genta and cisplatin) were treated with two concentrations of liposomal formulations (C1 = 6.25 µg/mL and C2 = 12 µg/mL). Drug (gentamycin 14 μg/mL and cis 7 μg/mL) and liposomal formulation exposure were performed simultaneously. After 48 h, cell viability was assessed using MTT, while mitochondrial membrane potential was investigated using JC-1 (see below).

**Figure 11:**
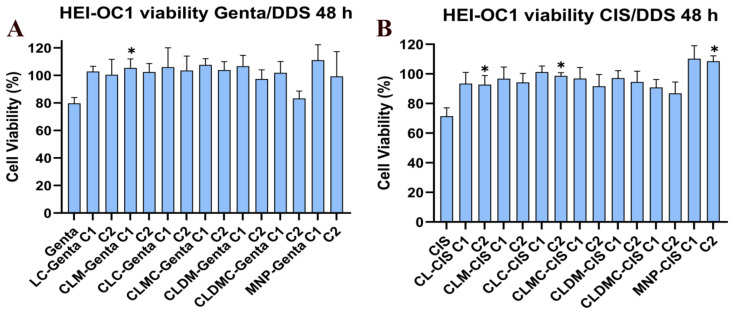
Cell viability of HEI-OC1 exposed to gentamycin (14 µg/mL) (**A**) and cisplatin (7 µg/mL) (**B**) and treated with two different concentrations (C1 = 6.25 µg/mL and C2=12 µg/mL) of liposomal formulations was assessed by MTT assay after 48 h, * = statistically significant; *p* ≤ 0.05.

Viability of treated cells (Figure 11) significantly increased compared to that of non-treated cells exposed to similar concentrations of genta and cis, respectively, for MNPs, CLM, and CLMC (in both concentrations used), while increasing non-significantly for CL and CLDM compared to controls (genta and cis-treated cells, respectively, without liposomes). Liposomal co-treatment increased cell viability under cisplatin and gentamicin exposure, highlighting their protective potential.

##### JC-1 Fluctuation in HEI-OC1 Exposed to Drugs and Treated with Liposomal Formulations

**Figure 12:**
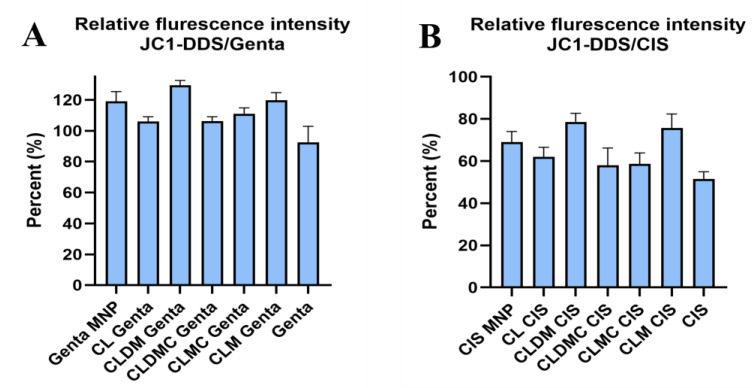
Relative fluorescence intensity of JC-1 fluorescent dye on HEI-OC1 cells shown after (**A**) gentamicin (14 µg/mL) or (**B**) cisplatin (7 µg/mL) exposure and treatment with liposomal formulations (12 µg/mL) for 48 h.

**Figure 13:**
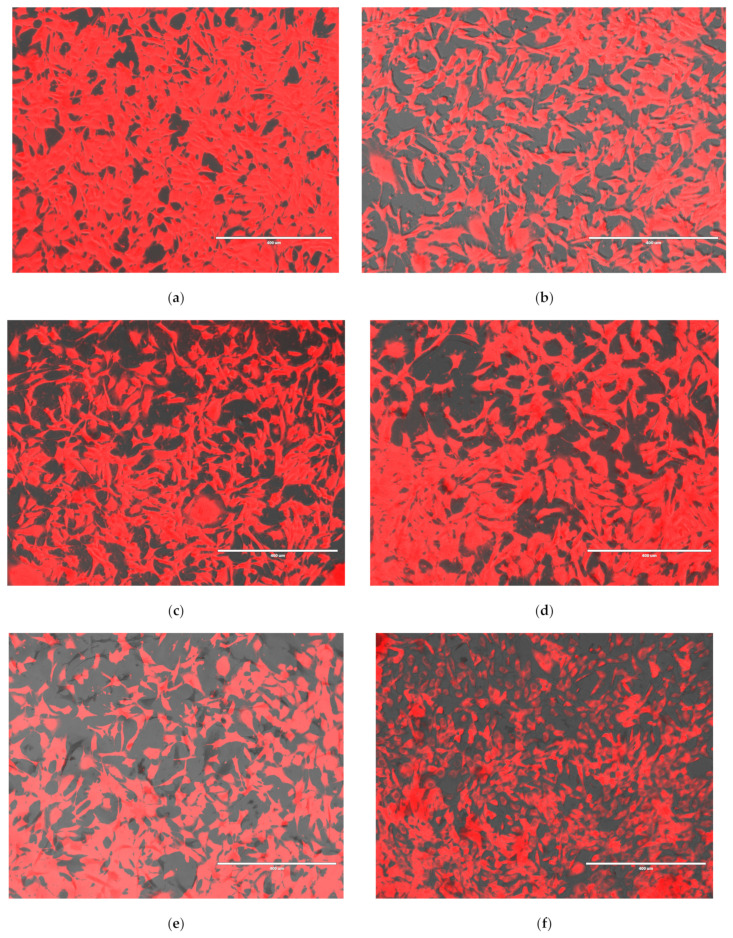

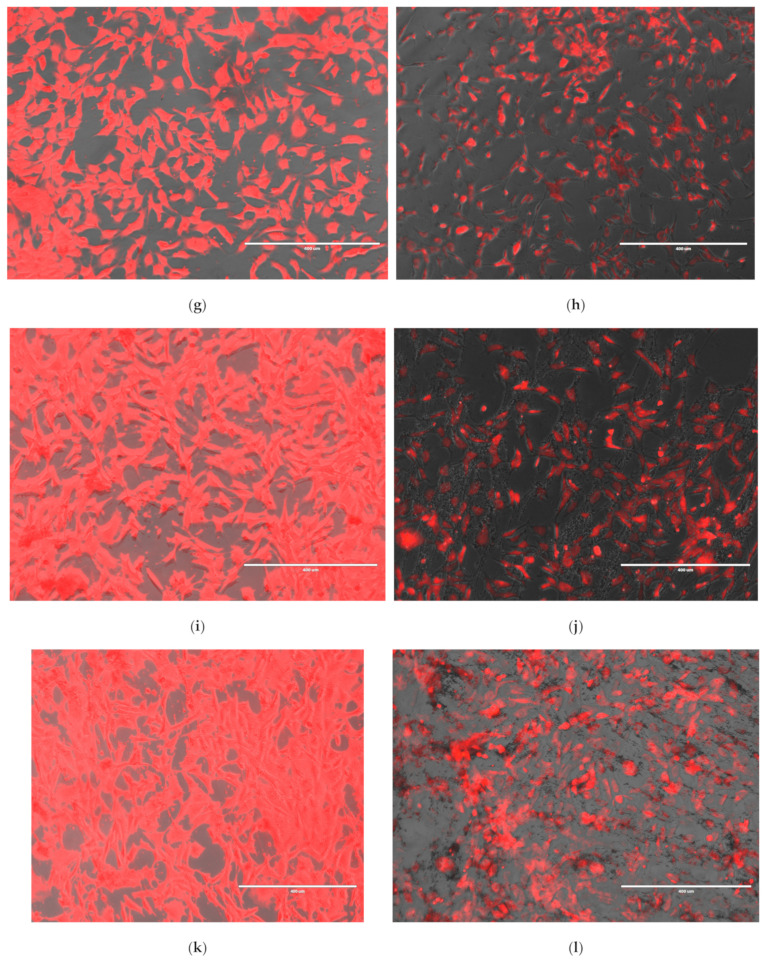

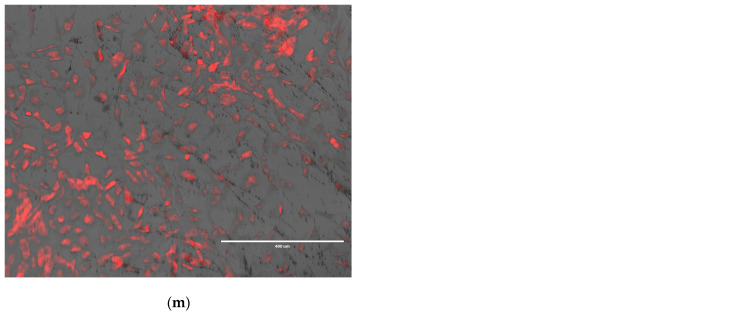
Fluorescence microscopy images of HEI-OC1 cells under gentamicin (14 µg/mL) and cisplatin (7 µg/mL) exposure treated with various liposomal formulations (12 µg/mL): (**a**) Cells—control; (**b**) cells—genta; (**c**) cells—genta/CL; (**d**) cells—genta/CLM; (**e**) cells—genta/CLDM; (**f**) cells —genta/MNPs; (**g**) cells—genta/CLDMC; (**h**) cells—cis; (**i**) cells—cis/MNPs; (**j**) cells—cis/CL; (**k**) cells—cis/CLM; (**l**) cells—cis/CLDM; (**m**) cells—cis/CDLMC. The photos represent overlay of bright field (BF), red fluorescence (RF), and green fluorescence (GF) channels obtained by fluorescence-inverted microscope EVOS magnification ×10, scale bar = 400 nm.

JC-1 has been developed as a fluorescent dye to monitor the loss of mitochondrial transmembrane potential (ΔΨ_M_). At high potentials, it forms red fluorescence J-aggregates (530 nm excitation and 590 nm emission) or green fluorescence J-monomers (490 nm excitation and 530 nm emission) for low MMP. Changing from red to green indicates a decrease in mitochondrial membrane potential commonly associated with apoptosis, since decreased ΔΨ_M_ reflects opening of mitochondrial permeability [22]. Cells that retain normal and functional mitochondrial membrane potential emit red fluorescence, while mitochondria with decreased ΔΨ_M_ emit green fluorescence. Images are detectable using an inverted fluorescent microscope. HEI-OC1 cells exposed to cis tend to emit predominantly green. Analyzing the ratio of red/green fluorescence on several FOVs using ImageJ enables detection of mitochondrial membrane potential fluctuations, calculated as percentage of non-treated, healthy controls. Notably, cis introduced significantly higher disturbances in mitochondrial membrane potential compared to gentamicin, decreasing the relative fluorescence intensity after 24 h exposure to almost 62% of the non-treated cells, while gentamicin treatment decreased it to 92.5%. Significant increases could be obtained by simultaneously exposing cells to CLDM (78.6%) and CLM (75.7%) for cis. Similar significant increases were obtained for genta by CLDM (129.6) and CLM (119.6), respectively, while MNPs, CLM, and CL increased the relative membrane potential non-significantly for both cis- and genta-exposed cells, as shown in Figure 12A,B and Figure 13. In the case of cis, a decrease and even a significant decrease in JC1 relative fluorescence intensity could probably be explained by the method of assessment of mitochondrial membranes using this test. Particularly, cis-treated cells tended to detach from their substrate. JC-1 quantitation relying on quantitative image assessment can be influenced by complete or even partial cell detachment.

Magnetic liposomes, especially drug-loaded variants, significantly restored mitochondrial function in drug-exposed HEI-OC1 cells.

##### Senescence-Associated Activity in HEI-OC1 Cells Treated with Gentamicin and Cisplatin Liposomal Formulations

**Figure 14:**
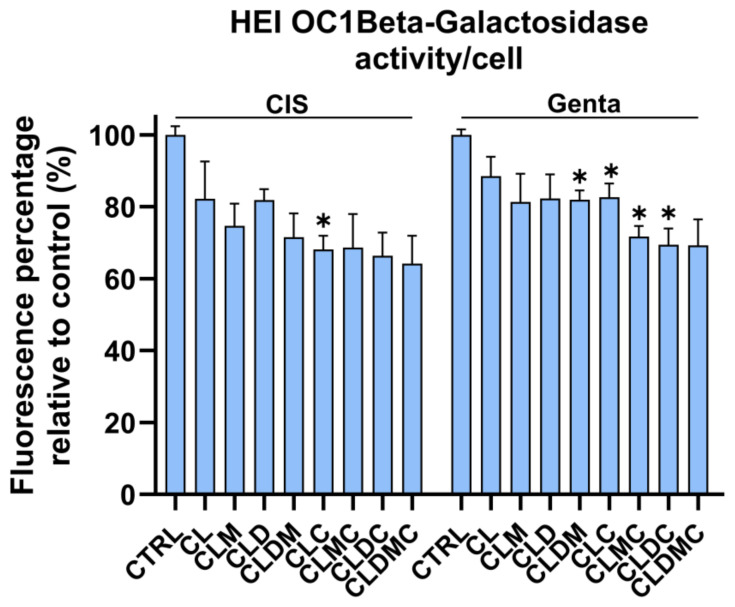
Senescence-associated beta-galactosidase activity of HEI-OC1 exposed to cisplatin (7 µg/mL) or gentamicin (14 µg/mL) treated with liposomal formulations (12 µg/mL) for 48 h, relative to drug exposed non-liposomal formulations treated controls; data are represented as mean and SD, *n* = 3; * *p* < 0.05; one-way ANOVA with Bonferroni correction vs. drug-only; * adjusted *p* < 0.05; ns, *p* ≥ 0.05.

Senescence-associated beta-galactosidase (Bgal) activity, detectable at pH 6.0, permits the identification of senescent cells in culture and in mammalian tissues. Bgal activity was tested in HEI-OC1 cells exposed to cis and genta, as well as in cells exposed to ototoxic drugs and treated with liposomal formulations after 48 h of cell–liposome interaction. Results were normalized to the number of cells detected by cell count in distinct wells. Compared to the cells untreated with liposomal formulations, and cis and genta exposed-controls, Bgal activity was significantly decreased in the cases of CLDMC treatment and non-significantly in the cases of CL, CLM, CLD, CLDM, and CLDC (Figure 14). Relative to drug-only controls, CLDMC produced a significant reduction in β-gal activity under both cisplatin and gentamicin exposure (adjusted *p* < 0.05), whereas CL, CLM, CLC, CLD, CLDM, and CLDC showed non-significant reductions (ns, *p* ≥ 0.05) (Table 3).

## 4. Discussion

The liposomal formulations proposed here are composed of cationic liposomes and magnetic cationic liposomes, uncoated or coated with CMCS, carrying a corticosteroid drug, Dex-P. Particularly after local administration (intratympanic injections), dexamethasone has shown protective effects against cisplatin-induced cochlear damage by preservation of outer hair cells and auditory function, based on ABR threshold shifts and hair cell rescue as detected by histological analysis. It reduces oxidative stress, inflammation, and apoptosis in cochlear hair cells by downregulating pro-inflammatory cytokines (e.g., TNF-α, IL-1β [41]). However, intratympanic administration produces an increased burst of local concentration, requires multiple injections, expertise, and carries procedural risks (e.g., tympanic membrane damage, infection). Besides, the short half-life in the inner ear produced by the rapid clearance of cochlear fluids limits sustained protective effects, necessitating repeated dosing or sustained-release formulations [42]. Therefore, smart drug delivery devices are needed to waive the need for invasive and repetitive local administration.

In recent years, several innovative approaches have emerged employing MNPs to enhance drug delivery to the inner ear—a key challenge in treating sensorineural hearing loss due to the blood–labyrinth barrier and limited cochlear access. For instance, intratympanic delivery of MNP-bound prednisolone, guided by external magnetic fields, achieved significantly improved protection of outer hair cells in mouse models of ototoxicity compared to conventional methods. More recently, MNP platforms have been specifically engineered for inner-ear delivery, using magnetic steering and laser-assisted activation to enhance payload penetration into the cochlea [43,44].

Liposome-based systems have many advantages in the controlled and targeted release of drugs and have also been used for the treatment of inner ear diseases [45,46]. These two types of systems (liposomes and MNPs) have been combined due to their advantages, with numerous studies showing the improvement of the drug release rate from liposomes to the target site by applying an external magnetic field [21,47,48]. However, in the specialized literature, we have not identified studies that present the use of this type of smart drug delivery system for the treatment of inner ear diseases. Our research group obtained and characterized a similar system, peptide-functionalized chitosan-based liposomes, for the treatment of inflammatory diseases [33]. The results obtained were promising, but the production cost increased due to the two peptides. For this reason, a new smart drug delivery system, magnetic liposomes coated with CMCS, was obtained. The preparation method for liposomes is not difficult and could easily be translated into large-scale production. Cationic liposomes coated with carboxymethyl chitosan encapsulating magnetic nanoparticles (MNPs) and a corticosteroid drug in the aqueous core were first characterized in terms of morphology, structure, stability over time, encapsulation, and release of the hydrophilic drug and magnetic properties. Following the analysis of the obtained results, a single ratio between the three constituent lipids (19/4/2 between EPC/Chol/DOTAP) was chosen to be tested in vitro in a drug-induced ototoxicity model. To highlight the efficiency of magnetic liposomes and CMCS-coated magnetic liposomes, precursor systems, cationic liposomes, and CMCS-coated cationic liposomes, unloaded or loaded with drug, were also tested in vitro.

The size of magnetic liposomes was not greatly influenced by the variation in lipid ratio, with the smallest size being recorded for the CLDM-3 sample, while the coating of magnetic liposomes with CMCS led to larger sizes; however, the smallest size was recorded for the CLDMC-3 sample. The zeta potential is an important characteristic that provides information about the stability of nanocarriers in aqueous media. Zeta potential values higher than 30 mV, in absolute value, indicate monodisperse suspensions without agglomerations, while values around ±20 mV are prone to have short stability, and those with values lower than 5 mV aggregate rapidly [49]. The zeta potential values that came close to 30 mV, in absolute value, were those in samples CLDM-3 (17.5 mV) and CLDMC-3 (20.4 mV) (see Table 2). These two samples were liposomes with a content of approximately 27% cholesterol in the composition. In the specialized literature there are studies that highlight the fact that liposomes obtained with a ratio of 30% cholesterol in the composition seem to be the most stable [50]. Therefore, these two samples have a similar amount of cholesterol in the composition (27%) and the zeta potential values recorded, compared to the other analyzed samples, could be explained accordingly.

Liposomes are lipid vesicle systems that are generally kept in suspension in an aqueous medium, and therefore, during storage, the encapsulated drug can be lost due to possible leakage [51,52]. Therefore, the stability of the obtained systems was tested at two different temperatures (refrigerator and room temperature). It was observed that for both tested temperatures, CMCS increased the stability of magnetic liposomes against leakage, and increasing cholesterol in the lipid mixture while improving their stability (Figure 3). The improved physical stability of liposomes when coated with CMCS contributes to the prolonged release of active substances, compared to uncoated liposomes [53]. In the present study, the best stability was demonstrated by the samples with CLDM-3 and CLDMC-3, which had 27% cholesterol in their composition (molar ratio).

The stability of CLDM-3 and CLDMC-3 formulations was tested under simulated in vitro conditions, 10% FCS medium, and in the other two media, ultrapure water and weak ionic medium, at 37 °C and 4 °C, for 48 h, focusing on three important parameters: size, PDI value, and zeta potential. In Figure 4 and Figure 5, it can be seen that the size of the analyzed samples depended on the exposure time, and the pH of the environment in which they were maintained, but also on the temperature used throughout the experiment. Therefore, it is found that the uncoated CLDM-3 liposomes, which were introduced into ultrapure water (pH ~ 7) at a temperature of 37 °C, had dimensions of approximately 265 nm, at 48 h from the start of the experiment (t_0_) (Figure 4A), and at a temperature of 4 °C they had dimensions of approximately 262 nm (Figure 5A). The coated liposomes had after 48 h a diameter of approximately 292 nm at a temperature of 37 °C and 270 nm at a temperature of 4 °C (Figure 4A and Figure 5A). The slight increase in the diameter of the CLDMC-3 liposomes, compared to CLDM-3, was expected due to the coating of the liposomes with CMCS.

In the case of tests in low ionic solution (pH ~ 7), an increase in the size of uncoated liposomes up to 690 nm at 37 °C and 662 nm at 4 °C was observed, which reveals a tendency for liposomes to agglomerate in this environment. This is also highlighted by the increased PDI value for the CLDM-3 sample (Figure 4B and Figure 5B). In the case of coated liposomes, the size after 48 h at 37 °C was 353 nm and 326 nm at 4 °C. Coating with CMCS led to an increase in the stability of liposomes in this environment, a fact demonstrated by the decrease in the PDI value (Figure 4B and Figure 5B).

Uncoated liposomes analyzed in 10% FBS medium had a diameter of approximately 700 nm after 48 h at 37 °C and 272 nm at 4 °C. In the case of the CLDMC-3 sample, the size at 37 °C was 739 nm, while at 4 °C the size was 517 nm. The increase in the diameter of the coated liposomes may be due to the attachment of proteins from the FBS medium to the liposome surface. These results are consistent with the relatively high PDI values (Figure 4B and Figure 5B).

The zeta potential values for liposomes coated with CMCS (Figure 4C and Figure 5C) were between −14 and −41.5 mV, which highlights an increase in their stability in all three environments analyzed, both at 37 and 4 °C.

Consequently, as a general conclusion, coating magnetic liposomes with CMCS led to an improvement in stability in terms of size and zeta potential in the aqueous media studied, with very few exceptions.

In vitro drug release tests showed that the drug was protected by the lipid membrane of the liposomes, and its release was gradual. The dialysis membrane delayed free drug passage, while encapsulated drugs showed slower release (4 h) (Figure 6a), and the drug encapsulated in magnetic liposomes and magnetic liposomes coated with CMCS recorded much lower proportions in this time interval. Thus, for the CLDM-3 sample, which had to cross the lipid membrane and then the dialysis membrane, after 4 h, only 25% of the loaded amount was released (Figure 6a). While for the CLDMC-3 sample, which in addition to the lipid membrane, was also coated with CMCS, the amount of drug released was much lower, almost 4% of the total loaded amount (Figure 6b). Over the next 20 h, the release rates increased, reaching 38% for the CLDM-3 sample (Figure 6a) and only 12% for the CLDMC-3 sample (Figure 6b). The degree of Dex-P release from the 3 CMCS-coated magnetic liposome samples recorded values of approximately 12%, with no notable differences between them (Figure 6b).

Magnetic liposomes were obtained to be used for drug delivery to the inner ear using magnetic field guidance. The magnetization curve of the CLDMC-3 sample demonstrated that CMCS-coated magnetic liposomes had superparamagnetic properties. This characteristic is particularly important in applications such as targeted drug delivery, magnetic resonance imaging (MRI), and biosensing, where precise control over the magnetic properties of particles is significant [21]. In addition, other properties such as biocompatibility or the possibility of being functionalized make them suitable for various therapeutic and diagnostic purposes.

All the systems proposed proved not to interfere with the viability of HEI-OC1 cells in the experimental culture conditions created. Cationic liposomes, however, resulted in the lowest cell viability, while the presence of magnetic nanoparticles conferred the minimum cytotoxicity and the highest percentage of cell viability. We have previously reported good viability results from similar liposomal formulation systems in a human dermal fibroblast cell line [32]. While HEI-OC1 cells are reportedly more prone to display decreased viability under exposure to a liposomal formulation [54], MNP incorporation proves to increase cell viability. Improved cell viability adds to the already mentioned MNP advantages regarding maneuverability in magnetic fields and traceability using existing experimental (such as particle imaging PI) or clinical imaging methods (MRI).

The ferrozine assay was performed in order to test quantitatively the amount of liposomal formulations that can be taken up by HEI-OC1 by simple addition within the culture media, as reflected by the iron content. The maximum amount of liposomes taken up was by CLDMC; however, all MNPs containing liposomal formulations produced an uptake of more than 5 pg/cell. These results are consistent with the literature pointing out that iron-based nanoparticles internalized by cells above 4, 4.5 pg/cell can be remotely actuated as well as tracked in vivo [55]. Therefore, simple administration of the proposed liposomal formulations could derive a method for remote-controlled delivery to the inner ear using external MF, a fact that needs to be confirmed in vivo in future animal model studies. Existent literature supports the fact that liposomal and magnetic liposomal formulation can safely deliver their therapeutic payload to inner ear compartments. Liposomal nanocarriers have been demonstrated to enter the inner ear safely without inducing auditory barrier disruption, inflammation, or apoptotic changes—implying biocompatibility with hair-cell regions [56]. Moreover, design parameters such as nanocarrier size, surface charge, and hydrophilicity are known to influence passage across the round window membrane and uptake by sensory cells [46].

In another study, magnetoresponsive liposomes—embedding magnetic components within the liposomal structure—were shown to offer enhanced localization potential via external magnetic fields, and improved imaging capabilities, providing a structural and functional rationale for magnetic targeting in the cochlea [57].

In this work, after proving that exposure to ototoxic drugs decreased the viability of HEI-OC1 in a direct relationship with the drug’s concentration, a model based on in vitro cells has been established to further test the effect of liposomal formulations. Remarkably, it was found that almost double the doses of gentamicin were needed to induce a similar decrease in HEI-OC1 viability compared to cisplatin. This reflects the higher ototoxicity of the cytostatic drug compared to the aminoglycoside antibiotic at the cellular level, reflecting the existing clinical experience.

In a subsequent step, healthy differentiated HEI-OC1 cells were simultaneously exposed to ototoxic drugs and several liposomal formulations. We have chosen concomitant exposure of HEI-OC1 cells to ototoxic drugs as well as to cationic liposomes since the potential therapeutic use for such formulation would preferably be preventive, administered either before or at least during ototoxic drug exposure. After 48 h of combined drug and liposomes exposure, cell viability, as assessed by MTT assay, was found to increase significantly for all tested liposomal formulations, except for with CL and CLDMC at 12 μg/mL, which showed an insignificant increase in viability compared to controls exposed to the drug alone. The better results obtained with lower doses in the particular case of CLDMC could possibly be explained by the slightly increased size of these CMCS-coated liposomes, which could impede contact with the cells at higher concentrations. Drug exposure induced morphological changes in HEI-OC1 cells, which tended to detach from the culture dish, potentially preventing interaction with larger-sized liposomes in higher quantities. Also, the amount of dexamethasone released from CMCS-coated liposomes during the test may have been insufficient to counteract the ototoxic effects induced by gentamycin and cisplatin.

Next, HEI-OC1 mitochondrial health and early apoptotic processes using JC-1 were tested, since the test allows for quantitative assessment of the mitochondrial polarization state, as well as changes in mitochondrial membrane potential that occur early in apoptosis. It was observed that HEI-OC1 exposed to ototoxic drugs decreased mitochondrial membrane potential compared to untreated cells, especially for cisplatin exposure (52%) and less in the case of genta (92%). All the tested liposomal formulations were able to rescue HEI-OC1 mitochondrial membrane potential by more than 100%. In the case of cisplatin exposure, the mitochondrial membrane increased significantly upon treatment with CLDM and CLM, while for all other formulations, the increase was less than complete restoration (100% of cells not exposed to the drug). Cisplatin is known to induce ototoxicity by disrupting mitochondrial function in auditory hair cells. Studies using zebrafish lateral-line hair cells have demonstrated that cisplatin exposure leads to mitochondrial hyperpolarization, increased mitochondrial calcium levels, and elevated reactive oxygen species (ROS) production. These mitochondrial disturbances are early events that precede caspase-3-mediated apoptosis in hair cells [56]. In mammalian models, cisplatin has been shown to activate caspase-9 and caspase-3 in cochlear hair cells, leading to apoptosis, maybe due to higher metabolic activity and increased mitochondrial calcium uptake in basal hair cells. [58]. Since mitochondrial dysregulation plays a critical role in cisplatin-induced ototoxicity, the significant restoration achieved using CLDM and CLM liposomes samples is very promising for the further development of new therapeutic solutions.

Exposure of HEI-OC1 to hydrogen peroxide (H_2_O_2_) has been previously reported to result in elevated β-gal activity, indicating senescence. Furthermore, therapeutic interventions have been shown to reduce β-gal activity in H_2_O_2_-treated cochlear cells, suggesting a mitigation of senescence [59,60]. In the present study, the β-gal enzymatic activity of HEI-OC1 exposed to ototoxic drugs simultaneously with the liposomal formulation was investigated. A significant decrease in enzymatic activity could be observed in the case of the CLDMC sample for both genta and cisplatin-treated cells, while the decrease, albeit not significant, could be observed for all other formulations. These findings confirm the protective effect of liposomal formulations by preventing drug-induced cellular senescence in HEI-OC1 cells. Although further studies are needed to confirm the results in vivo, the findings are promising and pave the way for the design of preventive and therapeutic methods that could circumvent drug-associated ototoxicity and subsequent hearing loss in patients receiving aminoglycoside antibiotherapy or cytostatic cocktails that include cisplatin.

While our findings indicate that CMCS-coated magnetic cationic liposomes exhibit promising protective effects against cisplatin- and gentamicin-induced cytotoxicity in HEI-OC1 cells, several limitations must be acknowledged. The potential interference of the liposomal components with optical assays and possible limited Dex-P release over 24–48 h highlight the need for interpretation within experimental conditions created. Further mechanistic investigation, as well as in vivo tests, will be needed to ascertain the biological pathways implicated and to determine the otoprotective effect in animal models of induced ototoxicity. A limitation of this study is the absence of a free dexamethasone control group in the in vitro assays, which would have allowed a direct comparison between the nanocarrier-delivered and free drug effects. Our focus was on evaluating the stability, cellular uptake, and protective properties of the liposomal systems themselves, given that the cytoprotective role of dexamethasone in ototoxicity models is already well established. Future work should include direct free-drug comparisons to further validate the added value of the proposed delivery platform. Nonetheless, the convergence of improved cell viability, mitochondrial membrane potential restoration, and reduced senescence in co-treatment models supports the feasibility of magnetically responsive, corticosteroid-loaded nanocarriers for inner ear protection. Further investigation is warranted to fully elucidate the therapeutic potential of this delivery platform.

In addition to targeted drug delivery to the inner ear via Eustache tube (ET) catheterization or through the external auditory canal using electromagnetic fields, in cases with residual hearing loss when cochlear implants (CI) are indicated, magnetic liposomes can be incorporated into the CI port electrode array, with the aim of delivering protective drugs, such as corticosteroids, directly into the cochlea or growth factors for auditory fibers in the spiral ganglion. Even in cases without residual hearing loss, magnetic cationic liposomes carrying protective or anti-inflammatory drugs can be incorporated into the IC electrode, aiming to minimize the effects of electrode insertion trauma.

## 5. Conclusions

The obtained results demonstrated that the tested liposomal formulations had a protective effect on HEI-OC1 cells by preventing cellular senescence induced by gentamicin and cisplatin. Also, our results suggest that these formulations can protect against drug-induced cytotoxicity, preserve mitochondrial function, and reduce senescence-associated activity. Taken together, these findings provide proof-of-concept evidence for the use of magnetically maneuverable, polymer-coated liposomal systems in inner ear drug delivery. While further studies are necessary to clarify the underlying mechanisms and confirm efficacy in vivo, this work lays the foundation for the development of targeted nanocarrier strategies for otoprotection. Although further in vivo studies are required, these results indicate that cationic liposomes have therapeutic potential and may help limit the need for animal experiments.

## Figures and Tables

**Table 1:** The experimental program for obtaining magnetic liposomes loaded with dexamethasone phosphate.

Sample Code	EPC/Chol/DOTAP (Molar Ratio)	EPC/Chol/DOTAP (mg/mg/mg)	Dexamethasone Phosphate (Dex-P) (mg)	MNPs (mg)	CMCS (1%)/ Liposomes Suspension (*v*/*v*)	Sonication Time (min)
**CMCS-Coated Magnetic Liposomes**						
**CLDMC-1**	1.00/0.18/0.05	22/2/1	25	12.5	1/1	80
**CLDMC-2**	0.95/0.18/0.10	21/2/2				
**CLDMC-3**	0.86/0.36/0.10	19/4/2				

For magnetic liposomes (CLDM-1, CLDM-2, and CLDM-3), CMCS was not used.

**Table 2:** Mean diameter, PDI values, zeta potential, MNPs, and dexamethasone loading efficiencies, and the final composition of the obtained magnetic liposomes. Data presented as mean ± SD, *n* = 5.

Sample Code	Mean Diameter (nm)	PDI	Zeta Potential (mV)	MNPs Loading Efficiency (%)	Drug Loading Efficiency (%)	The Final Composition (mg/mL of Each Component) EPC/Chol/DOTAP/MNPs/Dex-P/CMCS
CLDM-1	212.4 ± 7.2	0.47	14.5 ± 4.1	34.4 ± 1.6	9.2 ± 1.3	4.40/0.40/0.20/0.86/0.46/0.01
CLDM-2	210.2 ± 6.0	0.35	15.7 ± 1.1	31.7 ± 3.2	8.8 ± 0.8	4.20/0.40/0.40/0.79/0.44/0.01
CLDM-3	207.6 ± 8.2	0.30	17.5 ± 1.2	29.5 ± 1.2	8.4 ± 1.1	3.80/0.80/0.40/0.74/0.44/0.01
CLDMC-1	306.7 ± 7.6	0.28	−10.3 ± 0.2	34.4 ± 1.6	7.7 ± 1.9	4.40/0.40/0.20/0.86/0.39/0.01
CLDMC-2	272.3 ± 9.9	0.23	−11.2 ± 0.9	31.7 ± 3.2	7.1 ± 0.6	4.20/0.40/0.40/0.79/0.36/0.01
CLDMC-3	268.3 ± 5.8	0.32	−20.4 ± 1.6	29.5 ± 1.2	6.5 ± 0.4	3.80/0.80/0.40/0.74/0.33/0.01

**Table 3:** Summary of protective effects of liposomal formulations: cis—cisplatin; genta—gentamicin; CL—cationic liposomes; CLM—magnetic cationic liposomes; CLMC—CMCS-coated magnetic cationic liposomes; CLD—cationic liposomes with dexamethasone; CLDM—magnetic liposomes with dexamethasone; CLDC—CMCS-coated liposomes with dexamethasone; CLDMC—CMCS-coated magnetic liposomes with dexamethasone; MNPs—magnetic nanoparticles. ↑ increase; ↓ decrease; JC-1—mitochondrial membrane potential dye. Values represent relative changes compared to drug-only controls as derived from in vitro experiments.

Formulation	Cell Viability ↑ (%) vs. Drug Only	JC-1 Mitochondrial Potential (% of Control)	β-Gal Activity ↓ (Senescence)
MNPs	Significant ↑ (both cis and genta)	↑ non-significant	Non-significant ↓
Cationic Liposomes (CL)	Non-significant ↑	↑ non-significant	Non-significant ↓
Magnetic Cationic Liposomes (CLM)	Significant ↑ (best for cis)	Cis: ~75.7% ↑ significant; Genta: ~119.6% ↑ significant	Non-significant ↓
CMCS-Coated Liposomes (CLC)	Non-significant ↑	↑ non-significant	Non-significant ↓
CMCS-Coated Magnetic Liposomes (CLMC)	Significant ↑ (dose-dependent, better at low conc.)	↑, but less than full restoration at high conc.	Non-significant ↓
Cationic Liposomes + Dex (CLD)	Non-significant ↑	↑ non-significant	Non-significant ↓
Magnetic Liposomes + Dex (CLDM)	Significant ↑ (best for genta)	Cis: ~78.6% ↑ significant; Genta: ~129.6% ↑ significant	Non-significant ↓
CMCS-Coated Liposomes + Dex (CLDC)	Non-significant ↑	↑ non-significant	Non-significant ↓
CMCS-Coated Magnetic Liposomes + Dex (CLDMC)	Significant ↑ (esp. β-gal reduction)	↑ moderate; protective vs. both cis and genta	Significant ↓ (both cis and genta)

## Data Availability

The datasets generated during and/or analyzed during the current study are available from the corresponding author on reasonable request.

## Supplementary Materials

The following supporting information can be downloaded at: https://www.mdpi.com/article/10.3390/nano15191529/s1, Figure S1: (a) The intensity-weighted size distribution and (b) TEM image of magnetic nanoparticles.
