# Supplementary material for: Magnetic Cationic Liposomes-Based Delivery System Reduces Drug-Induced Cytotoxicity in an In Vitro Model of Hearing Loss
Source: Nanomaterials (Basel). 2025 Oct 7;15(19):1529. doi: 10.3390/nano15191529 (PMC12526436; doi:10.3390/nano15191529)
Supplement: Supplementary file 1 [file nanomaterials-15-01529-s001.zip › nanomaterials-3830717-supplementary.pdf]

Supporting Information

Magnetic Cationic Liposomes-Based Delivery System Reduces Drug-Induced Cytotoxicity in an In Vitro Model of Hearing Loss

Loredana Iftode [1,2,†], Camelia Mihaela Zara Danceanu [3,†], Anca Niculina Cadinoiu [4,*], Delia Mihaela Raţă [4], Marcel Popa [2,4,5,], Luminița Labusca [3,6,*] and Luminita Radulescu [1]

1. Faculty of Medicine, "Grigore T. Popa" University of Medicine and Pharmacy, 700115 Iasi, Romania; iftodeloredana@yahoo.com (L.I.); lmradulescu@yahoo.com (L.R.)
2. "Cristofor Simionescu" Faculty of Chemical Engineering and Environmental Protection, "Gheorghe Asachi" Technical University, 700050 Iasi, Romania; marcel.popa@guest.tuiasi.ro
3. National Institute of Research and Development in Technical Physics, 700050 Iasi, Romania; cdanceanu@phys-iasi.ro
4. "Ioan Haulica" Institute, Faculty of Medicine, "Apollonia" University of Iasi, 700511 Iasi, Romania
5. Academy of Romanian Scientists, 050044 Bucharest, Romania
6. Department of Orthopedics, "Sf. Spiridon" Emergency Clinical Hospital, 700111 Iasi, Romania

* Correspondence: anca.n.cadinoiu@univapollonia.ro (A.N.C.); drlluminita@yahoo.com (L.L.)
† These authors contribute equally to this work.

Contents

The intensity-weighted size distribution and TEM image of magnetic nanoparticles………..…..Figure S1

Morphology of magnetic nanoparticles

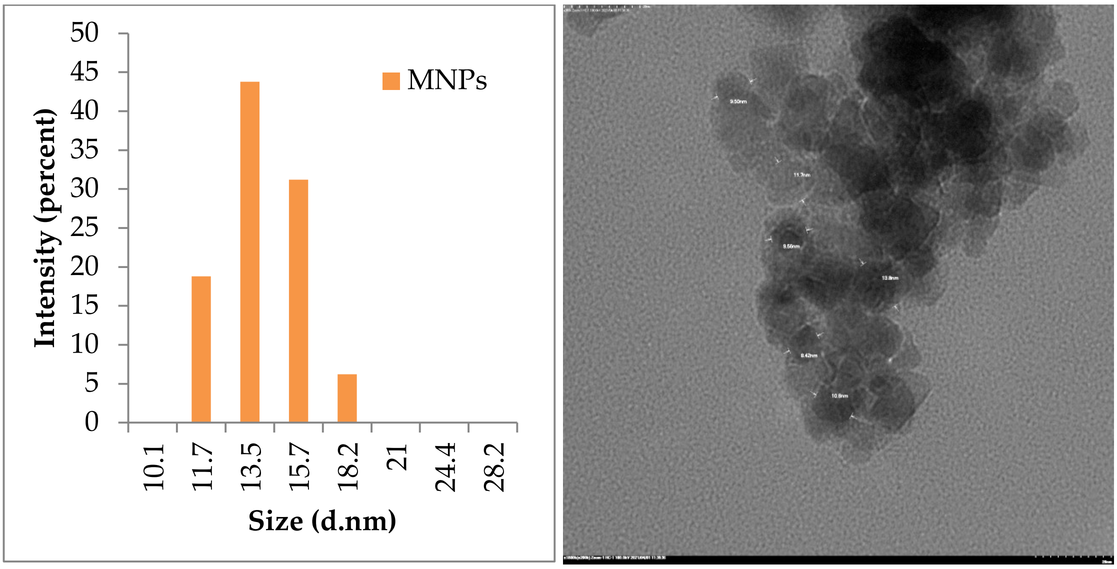

(a) (b)

Figure S1. (a) The intensity-weighted size distribution and (b) TEM image of magnetic nanoparticles

In the case of MNPs, the size distribution curve is monomodal with particle populations ranging between 10 and 21 nm (Figure S1 - a). The result obtained by TEM analysis (Figure S1 - b) reveals spherical MNPs with dimensions between 8.42 and 13.8 nm.

The zeta potential for magnetic particles in PBS (pH 7.4) had a value of -23.37±0.33 mV.

The lyophilization procedure

5 mL suspension of CMCS-coated magnetic liposomes were left in the freezer overnight and then transferred to the lyophilization chamber of the Freeze Dryer where it was maintained at a temperature of -50C and high vacuum for 48 h to remove water.
